# Improving Explainability and Integrability of Medical AI to Promote Health Care Professional Acceptance and Use: Mixed Systematic Review

**DOI:** 10.2196/73374

**Published:** 2025-08-07

**Authors:** Yushu Liu, Chenxi Liu, Jianing Zheng, Chang Xu, Dan Wang

**Affiliations:** 1 School of Medicine and Health Management, Huazhong University of Science and Technology Wuhan China; 2 Major Disciplinary Platform under Double First-Class Initiative for Liberal Arts at Huazhong University of Science and Technology (Research Center for High-Quality Development of Hospitals) Wuhan China; 3 Smart Hospital Research Institute, Peking University Shenzhen Hospita Shenzhen China; 4 School of Management, Hubei University of Chinese Medicine Wuhan China; 5 Hubei Shizhen Laboratory, Hubei University of Chinese Medicine Wuhan China; 6 Research Center for Traditional Chinese Medicine Development, Hubei University of Chinese Medicine Wuhan China

**Keywords:** artificial intelligence, explainability, integrability, healthcare professionals, systematic review

## Abstract

**Background:**

The integration of artificial intelligence (AI) in health care has significant potential, yet its acceptance by health care professionals (HCPs) is essential for successful implementation. Understanding HCPs’ perspectives on the explainability and integrability of medical AI is crucial, as these factors influence their willingness to adopt and effectively use such technologies.

**Objective:**

This study aims to improve the acceptance and use of medical AI. From a user perspective, it explores HCPs’ understanding of the explainability and integrability of medical AI.

**Methods:**

We performed a mixed systematic review by conducting a comprehensive search in the PubMed, Web of Science, Scopus, IEEE Xplore, and ACM Digital Library and arXiv databases for studies published between 2014 and 2024. Studies concerning an explanation or the integrability of medical AI were included. Study quality was assessed using the Joanna Briggs Institute critical appraisal checklist and Mixed Methods Appraisal Tool, with only medium- or high-quality studies included. Qualitative data were analyzed via thematic analysis, while quantitative findings were synthesized narratively.

**Results:**

Out of 11,888 records initially retrieved, 22 (0.19%) studies met the inclusion criteria. All selected studies were published from 2020 onward, reflecting the recency and relevance of the topic. The majority (18/22, 82%) originated from high-income countries, and most (17/22, 77%) adopted qualitative methodologies, with the remainder (5/22, 23%) using quantitative or mixed method approaches. From the included studies, a conceptual framework was developed that delineates HCPs’ perceptions of explainability and integrability. Regarding explainability, HCPs predominantly emphasized postprocessing explanations, particularly aspects of local explainability such as feature relevance and case-specific outputs. Visual tools that enhance the explainability of AI decisions (eg, heat maps and feature attribution) were frequently mentioned as important enablers of trust and acceptance. For integrability, key concerns included workflow adaptation, system compatibility with electronic health records, and overall ease of use. These aspects were consistently identified as primary conditions for real-world adoption.

**Conclusions:**

To foster wider adoption of AI in clinical settings, future system designs must center on the needs of HCPs. Enhancing post hoc explainability and ensuring seamless integration into existing workflows are critical to building trust and promoting sustained use. The proposed conceptual framework can serve as a practical guide for developers, researchers, and policy makers in aligning AI solutions with frontline user expectations.

**Trial Registration:**

PROSPERO CRD420250652253; https://www.crd.york.ac.uk/PROSPERO/view/CRD420250652253

## Introduction

### Background

The rapid development of artificial intelligence (AI) has demonstrated profound impacts across various industries, particularly in the health care sector. The application of AI has shown significant potential and is widely used in areas such as disease diagnosis, patient monitoring, robotic surgery, and clinical decision-making [1]. However, with the increasing prevalence of AI technology, issues concerning doctors’ acceptance, trust, and willingness to use AI have garnered widespread attention. A study revealed that only 10% to 30% of doctors use AI in real-world scenarios [2]. The poor acceptance and low use of AI systems by users are influenced by various factors, including the technical characteristics of AI itself, individual factors (eg, users’ AI literacy), organizational factors (eg, advocacy by management), and policy-related issues (eg, responsibility attribution in the use of AI). These challenges hinder the widespread adoption of AI technology [3-7].

Among the technical characteristics of AI, explainability and integrability are considered 2 key factors influencing doctors’ acceptance and use of AI [8,9]. While other elements, such as security and social influence, also play important roles in clinicians’ trust in AI [10,11], their practical impact on daily clinical adoption differs. Security concerns are essential for AI implementation, but are most often managed at the technical or regulatory level [10]. Social influence, encompassing peer and organizational advocacy, tends to influence adoption at the institutional level [12]. In contrast, multiple systematic reviews and clinician surveys have identified explainability and integrability as the most immediate and actionable factors influencing real-world AI adoption in health care [8-10,13-16]. Accordingly, focusing on explainability and integrability provides practical, user-centered insights for promoting effective integration of AI into clinical practice. In this study, *explainability* refers to the extent to which an AI system provides human-understandable and faithful representations of its decision-making process [13,14], while *integrability* refers to the extent to which AI systems can be embedded into clinical workflows with minimal disruption, ensuring usability, interoperability, and alignment with routine practices [10,15,16]. Importantly, integrability is a broader concept that encompasses interoperability. While interoperability ensures the technical capacity for systems to exchange and interpret data using standardized formats, integrability goes further to consider how AI systems align with clinical roles, decision-making contexts, workflow timing, and user experience [17,18]. A system may be technically interoperable but still lack integrability if it fails to deliver value in practice or imposes additional burdens on clinicians.

Explainability is crucial in the medical field, where decision-making is highly complex and involves significant risks. Clinicians need to ensure the accuracy and safety of AI outputs before they can trust and rely on AI. A lack of explainability in AI clinical decision support systems (AI-CDSS) may lead to distrust among decision makers and reduce their willingness to use these technologies [19]. In addition, integrability ensures that AI-CDSS can seamlessly integrate into existing clinical workflows. When AI-CDSS are effectively embedded into doctors’ daily routines, clinicians can more efficiently access and use patient data, receive recommendations that better meet practical needs, and reduce the time spent on redundant data entry, allowing them to focus on clinical decision-making [20]. Conversely, a lack of integrability in AI-CDSS may negatively impact clinician work by increasing operational complexity, workload, and time costs. Such systems, which fail to align with practical requirements, can reduce doctors’ willingness to adopt them [21-23].

However, on the one hand, most existing studies primarily neglect the end users, namely, physicians’ understanding of and need for explainable AI [24-27]. Studies have mainly concentrated on the perspectives of developers and researchers and have developed methods to present technical descriptions of model processes in AI [27], although such approaches seem meaningless if they are not aligned with physicians’ understanding of and requirement for explainability in real-world application in medical settings [26]. On the other hand, a few studies have explored how system compatibility, user-friendly design, and workflow adaptability [24-26] may contribute to the seamless integration of AI into the clinical workflow. However, what AI integrability entails and what its underlying components are from the physicians’ perspective remains unclear.

### Objectives

Therefore, this study aims to systematically review existing literature on explainability and integrability of AI systems in health care from the perspective of health care professionals (HCPs). Specifically, it seeks to identify how these 2 factors—explainability and integrability—influence clinicians’ acceptance and use of AI-based decision support systems. On the basis of the findings, the study proposes a conceptual framework that synthesizes key user-centered concerns, with the goal of informing the design of more acceptable and adoptable clinical AI tools.

## Methods

### Literature Search

We conducted this systematic review of systematic reviews according to a protocol registered in PROSPERO (CRD420250652253), and the databases we searched included PubMed, Web of Science, Scopus, IEEE Xplore, ACM Digital Library, and arXiv. We did not explicitly search proceedings from major machine learning and AI conferences such as Association for Computational Linguistics, Neural Information Processing Systems, International Conference on Learning Representations, or International Conference on Machine Learning, as many papers from these venues are concurrently available on arXiv. We used keywords such as “Explainable AI,” “explainability,” “XAI,” “AI integrability,” “usability,” and “human-centered AI” (the detailed search strategy is provided in Multimedia Appendix 1) and covered publications from January 2014 to July 2024. In addition, references cited in the retrieved articles and reviews were manually screened.

### Inclusion and Exclusion Criteria

Studies were eligible for inclusion if they addressed either the explainability or the integrability of AI in health care (ie, meeting at least one of the following two sets of topic-specific criteria): (1) explainability, the study must address at least 1 of the following aspects—how AI processes input information; how conclusions are reached; the rationale behind these conclusions; how explainability fosters user trust and their use; or the knowledge, attitudes, and perceptions of HCPs regarding AI explainability—and (2) integrability, the study must address at least 1 of the following aspects—how AI integrates with hospital information systems and clinical workflows; how integrability fosters HCPs’ trust and use; or the knowledge, attitudes, and perceptions of HCPs regarding AI integrability.

In addition, studies were required to (3) focus on the perspective of AI end users, specifically HCPs such as doctors, nurses, and medical laboratory staff and (4) be original research, including original quantitative, qualitative, or mixed methods studies.

Editorials, reviews, conference abstracts, narrative studies, and studies focusing on the perspectives of AI developers, engineers, or technical professionals were excluded (Textbox 1).

**Inclusion and exclusion criteria for the literature.**

**Inclusion criteria**

- Time: published between 2014 and 2024
- Population: artificial intelligence (AI) users, focusing on health care professionals (HCPs)
- Field: related to the medical field or broadly relevant to health care
- Outcome: the study must address at least 1 of the following aspects: explaining how AI processes input information; how AI reaches conclusions; the rationale behind AI conclusions; how AI integrates with hospital information systems or clinical workflows; AI usability; how explainability or integrability fosters user trust and use, HCPs (eg, doctors and nurses) knowledge, attitudes, or perceptions of AI explainability or integrability
- Study design: original qualitative, quantitative, or mixed method research
- Other: published in English

**Exclusion criteria**

- Time: published at other periods
- Population: AI experts, engineers, or other technical professionals
- Field: other specific industries
- Study design: abstracts, editorials, commentaries, reviews, and narrative studies
- Other: full text unavailable

To maximize comprehensiveness, we initially applied a broad search strategy, but studies were then screened against strict inclusion and exclusion criteria based on the study objectives. YL initially screened titles and abstracts to assess eligibility, with all decisions systematically recorded in a structured Microsoft Excel spreadsheet to ensure transparency. Owing to resource constraints, this stage was conducted by a single reviewer following strictly defined inclusion and exclusion criteria to minimize potential bias. For full-text screening, 2 reviewers (YS and CL) evaluated each study using a standardized Excel form designed to promote consistency in assessment. Any disagreements or uncertainties were discussed with a third author (JZ) until a consensus was reached, further strengthening the reliability of the screening process. Data extraction was conducted by a single reviewer but performed twice to ensure accuracy and completeness. Extracted items included study characteristics, AI system details, target health care settings, participant types, and key findings related to explainability and integrability. Any uncertainties during extraction were resolved by referring back to the full text.

To capture users’ perceptions of explainability and integrability across both health care and general domains, we initially adopted a broad search strategy without restricting to medical-specific terms. However, as 21 (95%) of the 22 included studies were from the health care domain, the final analysis focused on medical contexts.

### Quality Assessment

All included studies underwent quality assessment based on their respective study designs. Quantitative and qualitative studies were evaluated using the Joanna Briggs Institute critical appraisal checklists, while mixed methods studies were assessed using the Mixed Methods Appraisal Tool (version 2018). For the JBI checklists, studies scoring ≥80% (ie, meeting at least 8 of 10 items) were considered high quality, 60% to 79% as medium quality, and <60% as low quality. For the Mixed Methods Appraisal Tool, studies that met ≥4 of 5 criteria were rated high quality, while those meeting 3 were considered medium quality. Only studies rated as medium or high quality (22/26, 85%) were included in the final synthesis.

### Data Extraction and Evidence Synthesis

The data extraction process included the following items for each eligible article—(1) basic information: authors, year, study region, content, and participants; (2) methodology: study design, study population, data collection methods, and data analysis methods; (3) results: HCPs’ understanding and needs regarding AI explainability or integrability.

This study analyzed quantitative and qualitative results separately and integrated them through narrative synthesis, using a descriptive method to present the findings [28]. For qualitative data, we followed the guidelines of Braun and Clarke for thematic analysis. An inductive approach was applied to code data, covering 4 stages: familiarization with the data, initial coding, identification of themes, and review of themes. Thematic analysis was conducted using both deductive and inductive approaches. For the analysis of explainability, we adopted a deductive coding structure based on a preexisting conceptual framework, which includes 3 first-order dimensions: preprocessing, model-level, and postprocessing explainability [25]. Specifically, preprocessing explainability refers to enhancing transparency during data preparation and feature engineering before model training. Model explainability focuses on understanding and interpreting the inner mechanisms, parameters, or representations of the model itself during training. Postprocessing explainability involves applying interpretability techniques after model predictions, which can provide both local (instance-level) and global (model-level) explanations of the model’s behavior. These categories guided our initial coding and interpretation. Within each of these dimensions, we generated subthemes inductively from the data to capture participants’ nuanced perspectives. For integrability, no prior framework was applied; all themes were developed inductively based on participants’ responses. Because there is a lack of theory guiding the coding for integrability, themes regarding AI integrability emerged based on the coding of extracted text from retrieved studies. NVivo 10 was used for thematic analysis. YL conducted the initial coding individually, and the coded data were then reviewed by a second person to ensure the validity of the themes. Any discrepancies in coding were discussed in group meetings, where consensus was reached.

In addition, quantitative data related to the explainability and integrability of medical AI from the user’s perspective were also extracted. Owing to substantial heterogeneity in study outcomes and exposure measures, a meta-analysis was inappropriate, and a narrative synthesis was used to analyze the collated studies, including methods, sample size, participants, outcome variables, and dimensions of explainability and integrability.

## Results

### Literature Screening Results

**Figure 1:**
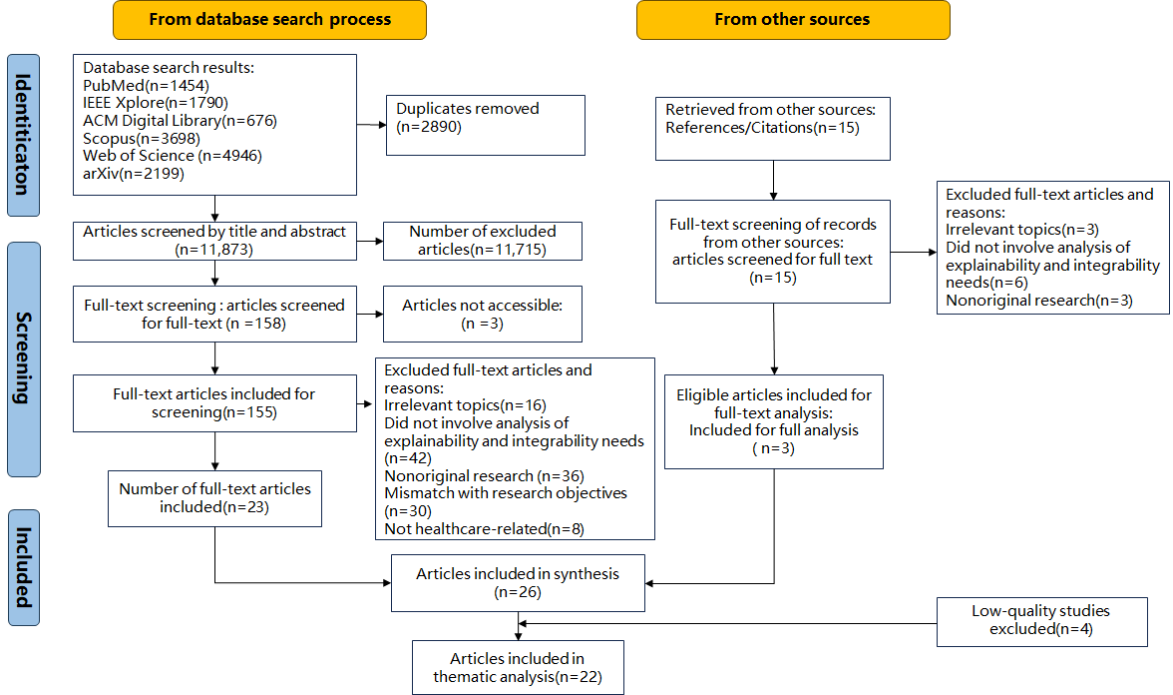
PRISMA (Preferred Reporting Items for Systematic Reviews and Meta-Analyses) flow diagram.

A total of 11,888 articles were retrieved through the search and their references. Among them, 26 (0.22%) articles met the inclusion criteria for this study. After quality evaluation, 4 (15%) low-quality articles were excluded, and a total of 22 (85%) articles were included in the analysis. The study selection process is shown in Figure 1.

### Characteristics of the Included Studies

All the included studies were published in 2020 or later. Most of the studies were conducted in developed countries (18/22, 82%), including 9 (41%) from the United States, and only 2 (9%) studies originated from developing countries (China and Brazil). Regarding study type, most studies (17/22, 77%) were qualitative in design, and 5 (23%) studies adopted quantitative or mixed method approaches. The basic characteristics of all the included studies are presented in Table 1.

**Table 1:** Characteristics of the included studies.

Study	Region	Study design	Content	Participants	Main findings
Graziani et al [13], 2023	Worldwide	Qualitative	Explainability	Health care professionals, industry practitioners, and academic researchers	Explainability in AI^a^ refers to the ability to understand and interpret how models make decisions. It is typically categorized into 3 types: intrinsic explainability, where models are inherently interpretable or designed to be transparent; global explainability, which focuses on understanding the overall behavior of a model through methods such as feature importance and visualization; and local explainability, which aims to explain individual predictions using techniques such as local approximations, counterfactual explanations, or adversarial examples.
Marco-Ruiz et al [29], 2024	Europe and America	Qualitative	Integrability	AI technology developers in hospitals, clinicians using AI, and clinical managers involved in adopting AI, among others	Interviewees highlighted that varying protocols across health care organizations can affect AI system effectiveness, emphasizing the need for local validation to assess performance and workflow impact. They also stressed the importance of tools such as process mining and visualizations to analyze patient pathways and optimize complex workflows using real-world data.
Liaw et al [23], 2023	Multicountry	Mixed method	Integrability	Clinicians managing diabetes	Interviewees expressed concerns that AI tools might affect patient outcomes and clinical workflows, leading to overdiagnosis, increased costs, and exacerbating health disparities and alert fatigue. The tool’s utility is limited for doctors familiar with their patients. Inaccurate data can cause false alarms or missed diagnoses, with a lack of evidence supporting its accuracy. A user-centered design is recommended to improve the system.
Panagoulias et al [30], 2023	Greece	Quantitative	Explainability	Medical personnel (including medical students and medical practitioners)	Clinicians rated diagnostic information, certainty, and related reasoning as very important, particularly when their diagnoses conflicted with AI recommendations
Wang et al [22], 2021	China	Qualitative	Integrability	Clinicians in rural clinics	Heavy workload: rural doctors handle many patients daily with frequent interruptions, leaving little time for detailed communication or documentation, hindering AI-CDSS^b^ use. Resource limitations: lack of necessary equipment and medications in clinics makes many AI-CDSS recommendations impractical, reducing their utility. Design mismatch: AI-CDSS are designed for time-consuming, standardized processes that do not fit the fast-paced rural practice, and poor integration with other systems leads to data and recommendation issues.
Zheng et al [31], 2024	America	Qualitative	Explainability and integrability	Pediatric asthma clinicians	User needs and challenges in using ML^c^ systems fell into 3 main areas: how well the system fits into daily workflows (eg, avoiding alert fatigue), the need for clear explanations behind system decisions, and difficulties adapting the tool to real-world settings.
Wolf and Ringland [32], 2020	America	Qualitative	Explainability	Users and developers involved in the design and use of XAI^d^ systems	Nonexpert users preferred simple, intuitive explanations of AI decisions, using clear language, visual tools, and real-life examples. They focused on fairness, transparency, and the impact on daily life, needing straightforward charts and simplified explanations to build trust.
Morais et al [33], 2023	Brazil	Qualitative	Explainability	Oncologists	Visualization helps: experts found visual elements useful for identifying major and minor influencing features. They also expressed a need for more detail: participants wanted more traceability to see how results are generated, enhancing confidence in decisions.
Helman et al [34], 2023	America	Qualitative	Explainability and integrability	Doctors, nurse practitioners, and physician assistants	Doctors primarily focused on several key aspects when using AI tools: analytic transparency, graphical explainability, the impact on clinical practice, the value of integrating dynamic patient data trends, decision weighting (how much to trust and balance AI outputs in real decisions), and display location—including usability and how the interface is viewed by patients and families.
Ghanvatkar and Rajan [11], 2024	Singapore	Quantitative	Explainability	Clinicians	The integration of XGB^e^ with SHAP^f^, as well as the combination of LR^g^ with SHAP, showed high usefulness because of their strong conceptual explanations, with the XGB and SHAP combination performing best in prediction but lowest in fidelity. Usefulness scores also improved during neural network training, indicating better alignment between explanation importance and predictive power over time.
Kinney et al [35], 2024	Portugal	Qualitative	Explainability and integrability	Doctors, educators, and students	Transparency in sources: users need to know where AI obtains its information to trust it, similar to how students cite sources. Impact on doctor-patient relationships: doctors fear AI could reduce personal interaction, mirroring issues with EHRsh. Increased burnout: additional AI-driven tasks may increase physician stress and lead to uncritical reliance on AI suggestions.
Burgess et al [36], 2023	America	Qualitative	Integrability	Endocrinology clinicians	The study proposes these design principles: (1) ensure algorithms are practical for clinical settings to avoid unrealistic insights; (2) allow clinicians to consider patient-specific factors and maintain control over model outputs; (3) avoid adding “research” tasks to patient visits; and (4) focus on aiding complex decisions in the workflow, not repeating known information.
Yoo et al [37], 2023	South Korea	Qualitative	Integrability	Medical and nursing staff in emergency departments and intensive care units of tertiary care hospitals	Anticipated benefits: most participants believe medical AI can reduce decision-making time and handle repetitive tasks, easing workloads and improving efficiency. Main concerns: worries include workflow disruptions, added tasks, reduced clinical autonomy, overreliance on algorithms, skill decline, alert fatigue, and the inability to integrate information beyond electronic records.
Schoonderwoerd et al [38], 2021	Netherlands	Quantitative	Explainability	Pediatrician clinicians	Diagnosis explanations are essential: clinicians agree that understanding how CDSS^i^ arrives at a diagnosis is important for trust and decision-making. Need for personalized explanations: while most information elements are seen as valuable, preferences vary, suggesting that explanations should be tailored to individual needs. Balance detail and overreliance: key explanation elements include evidence used, supporting or contradicting data, certainty level, missing information, alternative diagnoses, and past performance—but too much detail may lead to blind trust in the system.
Hong et al [39], 2020	America	Qualitative	Explainability	Practitioners in various industries, such as health care, software companies, and social media	Understanding model behavior: during validation, builders need to know why a model produces a specific output for a given case, especially when it performs unexpectedly. Methods such as LIME^j^ and SHAP help provide these insights. Feature importance analysis: builders assess model logic by examining feature importance, focusing not only on key features but also on less important ones to gain a complete understanding of decision-making.
Gu et al [16], 2023	America	Qualitative	Integrability	Medical professionals in pathology	Enhanced accuracy and efficiency: the xPath system improved diagnostic accuracy and efficiency, reducing workload and boosting confidence. Traceable evidence for transparency: it provides a layered evidence chain (eg, heat maps and confidence scores), making AI diagnoses transparent and verifiable. User-friendly design: the system aligns with pathologists’ workflow, supporting easy verification and adjustment of AI recommendations, thus enhancing usability and adoption.
Wenderott et al [40], 2024	Germany	Qualitative	Integrability	Radiologists	The key barriers to AI adoption are (1) workflow delays, (2) extra steps, and (3) inconsistent AI-CAD^k^ performance. The key facilitators are (1) good self-organization and (2) software usability.
Verma et al [41], 2023	Switzerland	Qualitative	Explainability and integrability	Clinicians involved in cancer care (large health care organizations)	Integration challenges: integrating AI into clinical practice is difficult because of issues with data integration, ontologies, and generating actionable insights. Trust and generalization: clinicians distrust “black-box” models, and AI performance varies across different populations, limiting widespread use.
Tonekaboni et al [42], 2019	Canada	Qualitative	Explainability	Clinicians in intensive care units and emergency departments	Transparency: doctors need to know the model’s context and limitations, such as missing patient information, to trust it even if accuracy is not perfect. Feature explanation: clearly explaining the features used in decisions helps build trust and guides appropriate use in different patient groups. Visualization: well-designed visualizations enhance understanding and support clinical reasoning.
Brennen [43], 2020	America	Qualitative	Explainability	End users and policy makers	Model debugging and understanding: XAI tools should help users understand model behavior (eg, using LIME or SHAP), but they often require advanced knowledge of ML. Bias detection: tools should identify and explain systemic bias in models and provide context to assess fairness and reliability. Building trust: clearly presenting the data and logic behind decisions helps users understand and trust AI systems.
Fogliato et al [44], 2022	America	Quantitative	Integrability	Radiologists	One-stage workflow boosts AI reliance: participants more closely followed AI suggestions, especially on noncritical points. AI outperforms but risks overtrust: AI performed better, but improvements were largely because of reliance on AI, even when incorrect. Workflow impacts experience: 1-stage users felt increased confidence and speed; 2-stage users found it more complex and burdensome.
Salwei et al [45], 2021	America	Qualitative	Integrability	Emergency physicians	The study identified 25 components for integrating a human factors–based CDSS^l^ into emergency departments, organized into 4 dimensions: time (when the CDSS is used), flow (how it integrates into workflows), patient journey scope (which care stages it covers), and level (integration at individual, team, and organizational levels).

^a^AI: artificial intelligence.

^b^AI-CDSS: artificial intelligence clinical decision support systems.

^c^ML: machine learning.

^d^XAI: explainable artificial intelligence.

^e^XGB: extreme gradient boosting.

^f^SHAP: Shapley additive explanations.

^g^LR: logistic regression.

^h^EHR: electronic health record.

^i^CDSS: clinical decision support systems.

^j^LIME: local interpretable model-agnostic explanations.

^k^AI-CAD: artificial intelligence–based computer-aided detection.

^l^CDSS: clinical decision support system.

### Qualitative Research

#### Dimensions of AI Explainability From the User’s Perspective

##### Overview

**Figure 2:**
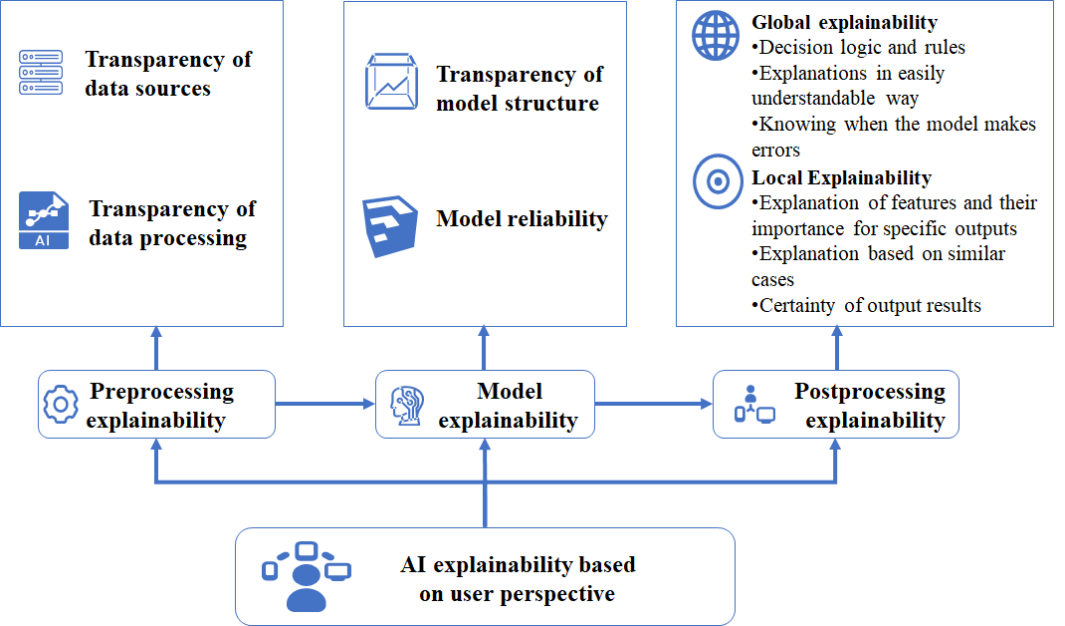
Conceptual framework of artificial intelligence (AI) explainability from the perspective of health care professionals.

A total of 16 articles focusing on the explainability of AI in the medical field were included [11,13,16,23,30-36,38,39,41,42,46]. According to the results of thematic analysis, HCPs are most concerned with postprocessing explainability, with 14 articles highlighting the necessity and importance of explanations provided in the postprocessing stage for HCPs [11,13,16,23,30-34,38,39,41,42,46]. The second most discussed aspect is the doctors’ concern regarding model explainability [23,31,42,46]. HCPs showed the least interest in preprocessing explainability [23,35,36]. The themes and subthemes regarding AI explainability from the user perspective are shown in Figure 2.

##### Postprocessing Explainability

###### Overview

Postprocessing explainability refers to the explanations provided after the AI system has made a decision or prediction. This stage focuses on clarifying the model’s output, helping HCPs understand how specific features or data points contributed to a particular decision. HCPs demonstrate the strongest interest in postprocessing explainability of AI, which could be divided into 2 dimensions: local explainability and global explainability. *Local explainability* refers to explanations for individual decisions or specific instances, helping HCPs understand how the model reaches a particular conclusion in a given situation. By contrast, global explainability refers to the HCPs’ understanding of how the model functions and its underlying decision logic. Comparatively, HCPs focus more on local explainability [11,13,16,23,31,33,34,38,39,41,42].

###### Local Explainability

On the basis of the current synthesis, local explainability is the most important aspect of explainability for HCPs, as highlighted by 12 studies, including (1) explanation of features and their importance for specific outputs, (2) certainty of output results, and (3) explanation based on similar cases.

First, the features of AI are the most critical component of local explainability, with 11 studies [11,16,23,31-34,38,39,41,42] highlighting that HCPs are concerned with which features were included and their importance in contributing to a specific AI-generated output. On the one hand, identifying which features were used in decision-making is fundamental for clinicians to build trust. By clearly showing the features used by the model, HCPs can verify their relevance, which enhances confidence in the model’s decisions. On the other hand, HCPs are also concerned with the extent to which a feature would impact an AI decision, and HCPs would construct trust if their perception of the importance of features was consistent with the results showing which features had the greatest impact on AI decisions [8,20], and visualization methods play a critical role in enabling HCPs to quickly grasp and understand how these features influence the predictions [31-33,39,42]. By providing clear visual cues such as color-coded severity indicators (eg, red, yellow, and green for high-, medium-, and low-risk categories, respectively) alongside numerical data, these visualizations allow HCPs to assess risk levels at a glance [31]. In addition, the ease of interpreting visual elements helps in distinguishing major and minor influencing factors [33], which enhances the accuracy and speed of decision-making in clinical settings. Moreover, personalized visualization designs are considered an effective way to enhance explainability [32]. Visualization schemes customized to meet HCPs’ specific needs can further improve their understanding of the model’s decision-making process. See illustrative descriptions from Zheng et al [31] and Hong et al [39]:

Visual indications of severity, such as red, yellow, and green to define high, medium, and low-risk categories paired with a numerical indication were required.

Zheng et al, 2024

Visual elements are easy to interpret and identification of minor influencing features...most domain experts acknowledged that the visual elements are easy to interpret and were able to perform the identification of major/minor influencing features.

Hong et al, 2020

Second, the certainty of output results also plays an important role in local explainability. Displaying the confidence or certainty of the model’s predictions provides HCPs with additional reference information, enabling them to better understand the model’s outputs and thereby increasing their trust in the model [38,42]. For example, providing CIs can help HCPs more effectively assess the reliability of the predictions [39]. See illustrative descriptions from Schoonderwoerd et al [38] and Hong et al [39]:

More specifically, clinicians stated that supporting- and counterevidence, and the certainty of the system will likely remain important in explanations, while information that is used to make the diagnosis, and the diagnosis in similar cases is likely to become less important over time.

Schoonderwoerd et al, 2021

Presenting certainty score on model performance or predictions is perceived by clinicians as a sort of explanation that complements the output result.

Hong et al, 2020

Third, AI local explainability means the model’s ability to explain its decisions by offering examples of previous instances that are similar to the current case [42]. This allows clinicians to interpret and understand how the model arrived at a decision based on prior cases with comparable features. It essentially helps in making the model’s predictions more transparent and interpretable through analogies to similar real-world examples. See illustrative description from Tonekaboni et al [42]:

For example, in cases where an ML model is helping clinicians find a diagnosis for a patient, it is valuable to know the samples the model has previously seen. Clinicians view this as finding similar patients and believe that this kind of explanation can be only helpful in specific applications.

Tonekaboni et al, 2019

In addition, the timing of explanations is a key consideration. Providing HCPs with excessive information may lead to information overload, potentially hindering their understanding of the system. For example, some participants noted that they do not want to verify every AI-generated result each time they use a clinical decision support tool. Instead, they only wish to delve into the underlying logic when the results are unexpected [36]. This indicates that HCPs prefer the ability to choose to access more information as needed, rather than being overwhelmed by excessive or redundant details during the explanation process.

A mixed method study confirmed the aforementioned results [38]. Schoonderwoerd et al [38] explored physician requirements for clinical support system explainability and surveyed 6 pediatricians, in which clinicians rated the following aspects as very important in any scenario involving AI-CDSS use, namely, features and their importance, results certainty, features increasing results certainty, and the ability of AI to generalize results to similar situations (median importance ratings were rated as highly important by doctors whether the diagnosis of the doctor and the computerized decision support systems is consistent).

###### Global Explainability

Six studies highlighted the importance of global explainability for HCPs [13,30,39,41,42,46], including (1) decision logic and rules, (2) explanations in an easily understandable way, and (3) knowing when the model makes errors.

First, explaining the decision logic and rules is a core part of understanding the overall functioning mechanism of AI models. This involves helping HCPs understand the entire decision-making process of the model, from input features to final outputs, including feature combinations, trade-offs, and the determination of decision boundaries (the dividing criteria set by medical AI). Such explanations are key to enhancing model transparency and enabling HCPs to grasp the overarching decision logic of the system [13,39]. See illustrative description from Hong et al [39]:

The majority of our participants desired better tools to help them understand the mechanism by which a model makes predictions; in particular regarding root cause analysis (P1), identification of decision boundaries (P3), and identification of a global structure to describe how a model works (P13).

Hong et al, 2020

Second, explaining the model in a way that is easy for HCPs to understand is also an important approach to enhancing overall explainability [39,42]. When the model’s explanations align with the HCPs’ cognitive models and logic, they serve as evidence to support decisions, which can greatly enhance the HCPs’ understanding and acceptance of AI model outputs. See illustrative description from Hong et al [39]:

Explanations for model predictions can be used as evidence (P16, P18, P20) to corroborate a decision, when ML model and user’s mental model agree.

Hong et al, 2020

Third, understanding when the model might make mistakes is another key aspect of global explainability. HCPs need to not only understand the normal decision-making logic of the model but also recognize the conditions under which the model may fail or generate incorrect decisions [13,39,42]. Only when HCPs are fully aware of the model’s limitations can they use AI cautiously in practical applications, thereby improving decision accuracy and safety. For example, HCPs should be informed of potential risks, such as when the model fails to account for specific historical data or lacks certain critical information [42].

##### Model Explainability

From the user’s perspective, the explainability of the model itself can be discussed regarding model reliability and the structural explainability of the model.

###### Model Reliability

Model reliability refers to the ability of an AI tool to consistently produce accurate and dependable results over time. It is typically assessed using performance metrics, such as accuracy, specificity, and sensitivity, which evaluate how well the model performs in predicting outcomes [23,31,42]. These metrics significantly influence clinicians’ initial adoption of the tools [42].

###### Transparency of Model Structure

Structural explainability, on the other hand, relates to how transparent and interpretable a model is in demonstrating the relationship between input features and the final output [42]. AI models based on algorithms, such as decision trees or logistic regression, can clearly demonstrate how input features influence the final output, helping clinicians understand the model’s parameters and reasoning mechanisms [46]. In contrast, “black-box” models, such as deep learning, while demonstrating superior performance in certain scenarios, have a complexity that makes it challenging for HCPs to understand their internal workings. See illustrative descriptions from Tonekaboni et al [42] and Fischer et al [46]:

Familiar metrics such as reliability, speciﬁcity, and sensitivity were important to the initial uptake of an AI tool, a critical factor for continued usage was whether the tool was repeatedly successful in prognosticating their patient’s condition in their personal experience.

Tonekaboniet al, 2019

If you know what that model is based on, it is not some mysterious black box where something comes out, but we as doctors know what those models are based on and what parameters are included. Then I can live with it not seeing the parameters for each prediction.

Fischer et al, 2023

##### Preprocessing Explainability

Preprocessing explainability refers to the transparency of AI systems when processing input data, including data sources and data preprocessing methods. Clinicians often need to understand what types of data AI systems are based on for predictions or diagnoses to evaluate the model’s applicability and reliability. This includes (1) transparency of data sources and (2) transparency of data processing.

First, the transparency of data sources is the foundation of HCPs’ trust. A total of 3 studies indicated that HCPs pay attention to the data sources of AI systems [23,35,36]. In the design of medical decision support systems, HCPs often want to know the origin of the data when they first encounter AI tools.

Second, the lack of transparency in data processing may undermine HCPs’ trust in AI systems. When HCPs cannot clearly understand how input data are processed, they may become skeptical of the system. For example, some HCPs mentioned that if they are unaware of who processes the input data, how they are processed, or how they are stored, this uncertainty can lead to reduced trust in the AI system [36].

#### Dimensions of AI Integrability From the User Perspective

**Figure 3:**
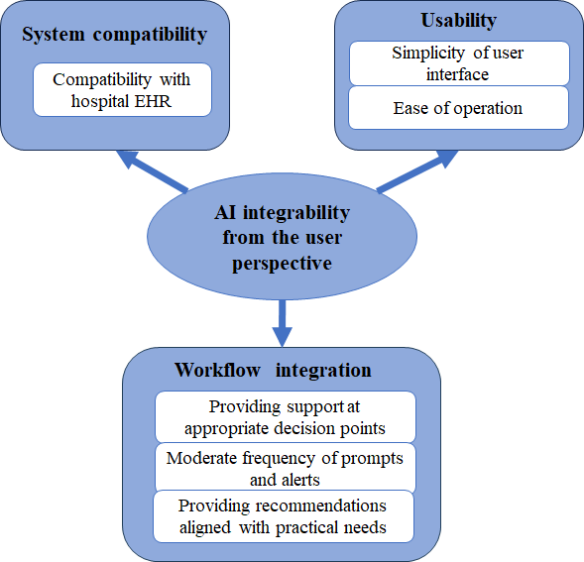
Conceptual framework of artificial intelligence (AI) integrability from the perspective of health care professionals. EHR: electronic health record.

According to the thematic analysis, AI integrability can be understood from the following 3 dimensions: workflow adaptation, system compatibility, and usability (Figure 3).

##### Workflow Adaptation

Workflow adaptation is a critical dimension of AI integrability, referring to the ability of AI systems to fit seamlessly into existing workflows without disrupting them, avoiding additional workload, and providing recommendations that meet practical needs [16,22,23,29,31,34-37,40,44,45]. This includes (1) providing support at appropriate decision points, (2) moderate frequency of prompts and alerts, and (3) providing recommendations aligned with practical needs.

First, the time points of AI assistance must not disrupt or interrupt doctors’ routines, increase workload, and extend time requirements for a workflow with well-integrated AI. It is essential to identify which complex decision points require AI assistance, rather than providing information or data that doctors already know [36]. This is especially problematic in high–patient-volume settings, where doctors are already under significant pressure. Workflow disruptions caused by AI systems can negatively impact various aspects of the diagnostic and treatment process [16,22,29,34-37,40,44,45]. See illustrative description from Wenderott et al [40]:

When using AI-CAD, Seven radiologists were concerned about potential time constraints associated with the software.

Wenderott et al, 2024

Second, attention must be paid to the frequency of AI alerts and notifications. Frequent alerts and information prompts from AI systems may lead to alert fatigue among doctors, making them desensitized to genuinely critical alerts. This can also result in information overload, further impairing doctors’ decision-making abilities [23,31,36]. See illustrative description from Zheng et al [31]:

In-basket message was mentioned by many clinicians as a common type of active alarm. However, it is necessary to balance effective information delivery and alert fatigue as clinicians, especially physicians, receive various alarms and notifications from multiple channels in their daily work.

Zheng et al, 2024

Third, 3 studies highlighted that it is crucial for AI outputs to align with the context of HCPs [22,23,29]. AI recommendations must match the operational capacity of HCPs and health care facilities, especially in resource-limited community clinics. For instance, if an AI system suggests conducting laboratory tests that cannot be performed or prescribing medications that are unavailable, it may reduce clinicians’ trust in the system and negatively impact its practical effectiveness [25]. Therefore, thorough local validation is essential when introducing AI systems to ensure they function appropriately within specific health care environments [29]. See illustrative description from Wang et al [22]:

In addition, since our research sites are first-tier community clinics, they are only capable of performing a limited number of laboratory examinations (e.g., none of the research sites have CT scan equipment). They also have very limited medication resources in stock. However, AI-CDSS would suggest a variety of laboratory tests, and treatment and medicine options, which clinicians often cannot prescribe. In this case, theses recommendations are often ignored by the clinician users.

Wang et al, 2021

##### System Compatibility

System compatibility is a critical dimension of AI integrability, particularly in the health care field, where it primarily refers to integration with electronic health records (EHRs). A total of 5 studies highlighted that many clinicians are willing to integrate AI systems with patient EHRs to provide more comprehensive and relevant information during clinical decision-making [23,31,35,37,40]. Integrating AI risk models into EHR systems can offer clinicians more valuable references, improving patient management while reducing repetitive tasks and minimizing information omissions in clinical workflows. See illustrative description from Kinney et al [35]:

Physicians cited the diverse factors that impact a treatment plan that is not able to be captured in an electronic system as a reason it may not be helpful.

Kinney et al, 2024

##### Usability

Usability refers to the ease with which users can interact with and effectively use a system to achieve their goals [47]. In the context of AI systems, usability is a key aspect, with 5 studies highlighting it as a critical factor that directly impacts the acceptance and effectiveness of the system in clinical settings [22,23,30,40,42].

###### Simplicity of User Interface

A user-friendly interface needs to be intuitive and easy to operate while providing timely and useful information without disrupting clinical workflows. The display and organization of interface functions are major dimensions of interface usability [22,40,42]. For example, one study found that overly frequent and space-consuming pop-up designs in clinical decision support systems hindered doctors’ access to other important information, leading to a poor user experience [22].

###### Ease of Operation

In addition, whether the system is easy for doctors to master directly affects its use. Clinicians tend to reject AI systems if they require significant time to learn [23,30]. A mixed method study conducted in diabetes management surveyed HCPs’ attitudes toward AI [23]. The results showed that 68% of participants considered usability (simple and easy operation) an important factor influencing their use. Another quantitative study, based on the technology acceptance model and diffusion of innovations theory, analyzed the key factors affecting the adoption of AI technologies among doctors and medical students, with 17.9% indicating the lack of user-friendly software and support systems as a barrier [30]. See illustrative description from Wang et al [22]:

A primary issue of AI-CDSS usability was that the system always pop up to occupy one-third of the screen, whenever the clinician opened a patient’s medical record in EHR. If the monitor’s screen size is small, the floating window of AI-CDSS may block the access to some EHR features (e.g., data fields). This frustrated many participants. To workaround this issue, clinicians had to minimize it while it was not in use.

Wang et al, 2021

### Quantitative Research

This study included 3 quantitative and 2 mixed method studies, focusing on HCPs’ willingness to use AI, the key influencing factors, explainability needs, and integrability. Only 1 quantitative study addressed AI integrability. Owing to the limited number of quantitative studies, they primarily serve to complement and validate the qualitative analysis in this study. See Table 2 for details.

**Table 2:** Characteristics of the publications of quantitative data.

Study	Research methods	Sample size	Participants	Outcome variables	Concerned dimensions of explainability or integrability
Liaw et al [23], 2023	Semistructured interviews and surveys	22	Clinicians managing diabetes	Factors influencing the adoption of the tool, perception of the tool’s usefulness, and ease of use	Transparency, usability, and impact on clinic workflows need to be tailored to the demands and resources of clinics and communities.
Schoonderwoerd et al [38], 2021	Domain analysis, interviews, surveys, and scenario experiment	6	Pediatrician clinicians	Diagnosis, information they have used in their decision-making, and the importance ranking of different types of explanations in various contexts	The information that is used to make a diagnosis, the information that supports the diagnosis, how certain the clinician is of the diagnosis, and the relevance of the information for their diagnosis
Panagoulias et al [30], 2023	Survey	39	Medical personnel (including medical students and medical practitioners)	Suggested level of explainability, knowledge of AI^a^, ways to better integrate AI, and AI concerns	The overall system functions, user-friendly software, and impact on workflow
Ghanvatkar and Rajan [11], 2024	Theoretical construction and case analysis	—^b^	Clinicians	Usefulness of AI explanations for clinicians	Local explanations and global explanations
Fogliato et al [44], 2022	Scenario experiment	19	Radiologists	Anchoring effects; human-AI team diagnostic performance and agreement; time spent and confidence in decision-making; perceived usefulness of the AI	Do not waste time and no additional workload.

^a^AI: artificial intelligence.

^b^Not available.

Two studies explored factors affecting physicians’ willingness to adopt AI, with a focus on explainability and ease of use. One mixed method study found that 77% of HCPs managing diabetes were willing to use AI, citing ease of use (68%) as a key factor [23]. Another study revealed that 25.6% of participants identified a lack of understanding of underlying technology as a barrier [30], which confirms the focus of HCPs on usability and explainability described in the previous section of the research.

Two studies focused on explainability needs. One found that post hoc local explanations, such as those provided by logistic regression and Shapley additive explanations (SHAP), received higher usability scores from clinicians than model-level explainability [11]. Another study found that clinicians rated diagnostic information, certainty, and related reasoning as very important, particularly when their diagnoses conflicted with AI recommendations [38]. *These quantitative results support and validate the qualitative findings that post hoc local explainability is crucial for HCPs.*

One study examined AI integration into workflows, comparing its placement in different stages of decision-making. It found that AI support at the start of a diagnostic session increased participants’ confidence and perceived usefulness but also highlighted that poor integration could increase task complexity and workload [44].

## Discussion

### Principal Findings

To enhance HCPs’ trust and use of AI-CDSS in future real-world clinical settings, this study adopted a mixed systematic review approach to synthesize evidence regarding AI explainability and integrability from the HCPs’ perspective. To the best of our knowledge, this study is the first to systematically summarize the concept of “AI integrability” from the HCPs’ perspective. It refers to the ability of AI systems to be easily and seamlessly integrated into workflows, providing timely, appropriately scaled, and practically relevant prompts or recommendations at the right points, without requiring excessive effort from the HCPs. HCPs’ needs for AI integrability are primarily reflected in 3 aspects: system compatibility, usability, and workflow adaptation.

Second, this study decodes the components of AI explainability based on HCPs’ lived experiences. It identifies that the core HCPs’ requirements of AI explainability can be divided into 3 stages, namely, data preprocessing, the AI model itself, and postprocessing explainability. Unlike the results from AI developers and researchers, the study found that general users (HCPs) are more focused on the explainability of the postprocessing stage, particularly local explainability, such as the importance of specific output features and the certainty of results. From the HCPs’ perspective, an explainable AI must clearly present data sources, processing workflows, model structures, decision mechanisms, and their rationales, using tools such as visualization and user-comprehensible language and logic to help HCPs understand and trust AI.

### Comparison With Existing Research

This study is the first to systematically review AI integrability from a comprehensive perspective. Current discussions about easily integrable AI only sporadically mention its compatibility with other systems and its ability to integrate into HCPs’ workflows (eg, the location of the AI and when it provides assistance), but lack in-depth and systematic exploration [48-50]. For instance, the study by Maleki Varnosfaderani and Forouzanfar [51] discussed the possibility of integrating AI with medical practice but did not thoroughly examine the specific needs faced by HCPs during the integration process. A study from rural clinics in China reported various tensions between AI-CDSS design and the rural clinical environment, such as misalignment with local environments and workflows, technical limitations, and usability barriers [22]. Another study concerning AI-CDSS in emergency departments identified integrability factors (eg, time, treatment processes, and mobility) through interviews with 12 emergency department doctors, but it was limited to a specific environment with a small sample size [45], resulting in poor extrapolation capability.

This study proposes, from the perspective of HCPs’ needs, that AI explainability should not only focus on technical transparency but also emphasize HCPs’ understanding and trust, particularly in clinical settings, where AI explanations should support HCPs in making more accurate and effective decisions. Existing research in explainable AI primarily focuses on algorithms, with related reviews mainly discussing taxonomies of explainability and technological innovations [25,52]. For example, Markus et al [53] proposed an explainability framework that mainly focuses on providing better tools for developers but largely explores explainability from an algorithmic perspective. Similarly, Amann et al [14] highlighted ethical and technical issues in explainable medical AI, pointing out the multidimensional nature of explainability, but their research remains focused on algorithm optimization and technical compliance. This study emphasizes the user perspective, offering more practical guidance for the design and promotion of explainable medical AI. The study found that HCPs focus more on postprocessing local explainability, meaning how specific predictions made by the model can explain changes in the patient’s condition or decision-making basis. This aligns with Shin [54], who emphasized that local explainability and causal relationships are key to user trust. A systematic review from medical and technical perspectives also supports this view [55]. Unlike data experts, who focus on model and data-layer explainability [25], users prioritize post hoc explainability. Some experimental research shows users (eg, doctors) have a higher understanding and acceptance of post hoc explanations, which are more actionable than traditional technical explanations [56,57]. This preference stems from 3 key clinical needs. First, post hoc local explanations help clinicians understand AI predictions in the context of individual patient cases, enabling more personalized and relevant decision-making [54]. Second, given their professional and legal responsibilities, doctors need to justify their choices based on understandable and traceable reasoning. Post hoc explainability provides the transparency required to assess whether AI outputs align with clinical guidelines and ethical standards [53]. Third, in high-pressure clinical environments, HCPs prioritize usability over theoretical clarity. Local, case-specific explanations are more practical and immediately applicable, which enhances trust and facilitates integration into routine workflows [58]. Thus, this study’s conclusion better matches real clinical scenarios, offering insights for developers to create AI-CDSS that meet the needs of HCPs.

### Challenges for Explainability and Integrability

Despite these advances, significant challenges remain in achieving explainability and integrability of AI-CDSS in clinical practice. These challenges are as follows.

#### Lack of Tailored Explainability Methods for HCPs

A key challenge is the lack of explainability methods tailored to HCPs [59,60]. HCPs focus on the explainability of the postprocessing stage, especially local explainability. To address this, post hoc explainability techniques such as local interpretable model-agnostic explanations (LIME) and SHAP [61] explain decision-making in black-box models, helping HCPs understand how predictions are made based on input data. For instance, Alabi et al [62] demonstrated the use of SHAP and LIME in prognostic modeling for nasopharyngeal carcinoma, highlighting their potential in clinical decision support. Simplifying output by avoiding technical jargon and using graphical explanations [63] allows HCPs to adjust detail levels to avoid overload. Medical AI can also offer personalized explanations based on roles, preferences [64], or feedback after explanations [65].

#### Dynamic Nature of AI Models Affecting Explanation Consistency

A significant challenge in explainable AI for clinical use lies in the evolving nature of explanations as AI models are continuously updated. These updates—whether for improving performance, incorporating new data, or aligning with emerging medical knowledge—can change a model’s internal logic, rendering previously valid explanations obsolete or misleading [66]. In clinical settings, where trust and transparency are paramount, outdated explanations may lead to incorrect interpretations or reduced confidence in AI recommendations. To address this, explainability methods must be adaptive—capable of automatically regenerating explanations following model updates, tracking changes over time, and surfacing the rationale behind those changes [67]. Therefore, maintaining the temporal validity of explanations is as crucial as ensuring their initial explainability, especially as AI systems become increasingly dynamic and responsive to new clinical evidence.

#### Limited Technical Compatibility With Existing Information Systems

Compatibility with existing information systems is a major challenge [68]. AI-CDSS often require large amounts of patient data to provide decision support, but if these data cannot be electronically retrieved, clinicians must manually input them, leading to frustration and abandonment [69]. Integrability difficulties are also linked to the lack of semantic interoperability standards [70]. To address this, standardized application programming interface and data format protocols should be developed to enable AI systems to automatically retrieve patient data from EHRs, reducing manual workload. In addition, implementing standards such as the Fast Healthcare Interoperability Resources and Unified Medical Language System can facilitate integrability with various patient information systems [71,72].

#### Complexity of AI Integrability in Clinical Settings

While this review identifies key enablers of AI integrability—namely, system compatibility, usability, and workflow adaptation—it is important to emphasize that integration is rarely seamless in real-world clinical settings. These dimensions, although essential, do not guarantee smooth adoption. For example, perceptions of usability often vary among different clinical roles, leading to inconsistent engagement [73]. Embedding AI tools into existing workflows can require significant adaptation, redefinition of tasks, and role negotiations. Even when systems are technically compatible, they may introduce new tensions, including resistance from clinicians or disruption to established routines [22]. Thus, integrability should not be treated purely as a technical process, but as a complex challenge shaped by cultural norms, institutional readiness, and professional autonomy [16,40,74].

To address this, frequent involvement of HCPs during system design, continuous feedback loops, and adaptation to local workflows are crucial. Methods such as human-computer interaction with expert input [75] and consumer journey mapping [76] have been used to enhance AI-CDSS integration. At the same time, it is necessary to develop a standardized diagnostic support framework that aligns AI with specific clinical needs [77]. Another promising direction to enhance integrability is dynamic adaptation, where AI-CDSS adjust their level of support based on contextual factors such as patient volume, emergency status, or resource availability [22,78]. In high-pressure situations (eg, during emergencies or when clinician workload is high), the AI system could provide more proactive or autonomous recommendations. Conversely, during low-acuity periods, it could take a more supportive or background role, allowing clinicians greater control. Such adaptability can reduce disruption, improve acceptance, and ensure that AI interventions align with real-time clinical needs and capacities.

#### Ethical Concerns

In addition, the ethical implications of explainability and integrability should not be overlooked. Explainability is ethically significant in supporting informed consent, accountability, and clinicians’ ability to critically evaluate AI recommendations [14]. When clinicians can understand how an AI system reaches its conclusions, they are better equipped to maintain professional autonomy and protect patient rights. Integrability also has ethical implications. If an AI system is not well integrated into clinical workflows, clinicians may not know when or how to use it properly. This can create confusion about who is responsible for decisions influenced by AI. For example, if an AI recommendation appears at the wrong time in the workflow or is difficult to interpret in context, a clinician might follow it without full understanding or ignore it when it should have been considered. In both cases, the boundaries of responsibility become blurred [22,79]. These issues highlight the necessity of designing AI systems that align not only with technical and operational requirements but also with core ethical principles, such as transparency, fairness, and trustworthiness in health care.

### Implementation Strategies Based on the Exploration, Preparation, Implementation, and Sustainment Framework

As noted in the previous section, AI faces persistent challenges in explainability and system integration. These cannot be resolved through isolated interventions but require a structured, phased approach. The exploration, preparation, implementation, and sustainment framework—comprising exploration, preparation, implementation, and sustainment—offers a widely validated model for supporting health care technology adoption and enhancing clinician acceptance of AI systems [80]. To systematically address these challenges, we draw on the exploration, preparation, implementation, and sustainment framework, providing structured strategies for each phase of implementation.

In the exploration phase, institutions should identify clinical needs and collaborate with multidisciplinary teams (eg, physicians, nurses, and IT staff) to assess AI integration opportunities. Prioritizing explainability, especially alignment with clinical reasoning, is critical. Models supporting SHAP, LIME, or other visual local explanation tools are recommended, alongside qualitative feedback collection from end users [81].

The preparation phase focuses on resolving integration barriers and tailoring explanations for different roles. Multilevel explanation interfaces can accommodate varying expertise levels [32,57]. Close coordination with IT departments is essential to embed AI tools into existing EHR systems, minimizing manual input and workflow disruption [71,72]. Organizational readiness and role-specific training are also key to successful adoption [82].

In the implementation phase, a phased deployment strategy helps minimize disruption and support gradual clinician adaptation. AI-CDSS tools can first target low-risk, supportive tasks (eg, risk alerts and abnormal laboratory flagging), then gradually expand to core decision-making such as diagnosis or treatment support. To ensure alignment with clinical needs, implementation should combine performance metrics (eg, alert response times and override rates) with user feedback (eg, satisfaction ratings and suggestion boxes). Regular log reviews and focus groups can surface usability issues and guide iterative improvement [83].

The sustainment phase focuses on the long-term integration of AI into clinical workflows. Continuous monitoring of system performance and user experience is essential to ensure sustained adoption [84]. As models evolve, transparent update mechanisms—such as automatically generated explanation revisions and change logs—should be maintained to support clinician trust and promote continued engagement with the system.

### Strengths and Limitations

This study offers a systematic exploration of AI integrability from the HCPs’ perspective, providing a conceptual framework to guide medical AI design and development. Unlike previous studies focusing on technical developers or researchers’ perspectives [85,86], it emphasizes the needs of actual HCPs, such as physicians. It establishes an AI explainability framework based on their priorities in data preprocessing, model structure, and postprocessing. This approach facilitates the development of user-centered AI-CDSS, promoting its acceptance and use by HCPs.

The limitation of this systematic review is the limited number of quantitative studies, which restricts quantitative analysis and statistical inference. Future research should include high-quality quantitative studies to validate and complement the conclusions.

Although we used a comprehensive set of keywords, the decision not to use truncation (eg, an asterisk) may have led to the omission of some relevant studies. This choice was made to maintain specificity, but it may have limited the search breadth. To address this, we supplemented the search with citation tracking. This limitation is acknowledged in the review to clarify the scope of our search strategy.

In addition, while emphasizing user perspectives, this review provides limited analysis of varying needs among different medical roles. This is partly because of the small number of eligible studies and the lack of detail regarding specific clinical tasks, settings, or user groups in many of the included papers. These limitations made it difficult to conduct deeper, context-sensitive analysis of HCPs’ perceptions of explainability and integrability. In this regard, we recommend that future research draw on implementation frameworks such as the Consolidated Framework for Implementation Research or Promoting Action on Research Implementation in Health Service framework to better account for the contextual and role-specific factors that shape HCPs’ experiences. These frameworks can support more nuanced analyses of clinical settings and tasks, guiding the development of AI tools that are better aligned with real-world practices.

### Conclusions

In conclusion, the explainability and integrability of medical AI are key factors influencing its acceptance and use in clinical settings. On the basis of the user-centered conceptual framework proposed in this study, future AI design should focus on HCPs’ needs to enhance explainability and integrability, thereby promoting HCPs’ acceptance and use and improving its effectiveness in real-world clinical applications.

## Appendix

Multimedia Appendix 1 Search strategy for artificial intelligence (AI) explainability and for AI integrability from the user perspective.

Multimedia Appendix 2 PRISMA (Preferred Reporting Items for Systematic reviews and Meta-Analyses) checklist.

## Abbreviations

AI: artificial intelligence
AI-CDSS: artificial intelligence clinical decision support systems
EHR: electronic health record
HCP: health care professional
LIME: local interpretable model-agnostic explanations
SHAP: Shapley additive explanations

## Data Availability

The data used and analyzed in this study will be made available by the corresponding author upon reasonable request.
