# Supplementary material for: Improving Explainability and Integrability of Medical AI to Promote Health Care Professional Acceptance and Use: Mixed Systematic Review
Source: J Med Internet Res. 2025 Aug 7;27:e73374. doi: 10.2196/73374 (PMC12371287; doi:10.2196/73374)
Supplement: Multimedia Appendix 1 [file jmir_v27i1e73374_app1.docx]

**Appendix 1**

Table 1. Search Strategy for AI Explainability from the User Perspective

| **Search Strategy** | **Number** | **Time** | **Database** |
| --- | --- | --- | --- |
| (("Explainable AI"[Title/Abstract]) OR ("Interpretable AI"[Title/Abstract]) OR ("transparency"[Title/Abstract]) OR ("explainability"[Title/Abstract]) OR ("explainability"[Title/Abstract]) OR ("integrability"[Title/Abstract]) OR ("interpretable AI"[Title/Abstract]) OR ("usability"[Title/Abstract]) OR ("human-centered AI"[Title/Abstract])OR ("XAI"[Title/Abstract])) AND ((accept[Title/Abstract]) OR (adopt[Title/Abstract]) OR (use[Title/Abstract])) AND (("AI"[Title]) OR ("machine learning"[Title]) OR ("Artificial Intelligence"[Title])) AND ((fft[Filter]) AND (2014:2024[pdat])) AND (fft[Filter]) | 977 | 2024/6/11 | pubmed |
| ((("Abstract":"Explainable AI"OR "Interpretable AI" OR "Abstract":"transparency" OR "Abstract":"explainability" OR "Abstract":"explainability"OR  "Abstract":"interpretable AI" OR "Abstract":"usability" OR "Abstract":"human-centered AI" OR "Abstract":"XAI") AND ("Abstract":"acceptance" OR "Abstract":"adoption" OR "Abstract":"use") AND ("Abstract":"machine learning" OR "Abstract":"AI" OR "Abstract":"Artificial Intelligence"))) | 1324 | 2024/6/11 | IEEE Xplore |
| Abstract:("Explainable AI" OR "Interpretable AI" OR "transparency" OR "explainability" OR "explainability" OR "integrability" OR "interpretable AI" OR "AI usability" OR "human-centered AI" OR "XAI") AND Abstract:("accept" OR "adopt" OR "use") AND Abstract:("AI" OR "machine learning" OR "artificial intelligence") | 457 | 2024/6/11 | ACM Digital Library |
| ( TITLE-ABS-KEY ( "explainable ai" ) OR TITLE-ABS-KEY ( "Interpretable AI" ) OR TITLE-ABS-KEY ( transparency ) OR TITLE-ABS-KEY ( explainability ) OR TITLE-ABS-KEY (explainability ) OR TITLE-ABS-KEY (integrability ) OR TITLE-ABS-KEY ( "interpretable AI" ) OR TITLE-ABS-KEY (usability ) OR TITLE-ABS-KEY ( "human-centered AI" ) OR TITLE-ABS-KEY ( "XAI" ) ) AND ( TITLE-ABS-KEY ( "accept" ) OR TITLE-ABS-KEY ( "adopt" ) OR TITLE-ABS-KEY ( "use" ) ) AND ( TITLE-ABS-KEY ( "AI" ) OR TITLE-ABS-KEY ( "artificial intelligence" ) OR TITLE-ABS-KEY ( "machine learning" ) ) PUBYEAR > 2014 AND PUBYEAR < 2024 AND PUBYEAR > 2014 AND PUBYEAR < 2024 | 654 | 2024/6/12 | Scopus |
| (AB=(Explainable AI) OR AB=(Interpretable AI) OR AB=(transparency) OR AB=(explainability) OR AB=(explainability) OR AB=(integrability) OR AB=(interpretable AI) OR AB=(usability) OR AB=(human-centered AI) OR AB=(XAI)) AND (AB=(accept) OR AB=(adopt) OR AB=(use)) AND (TI=(AI) OR TI=(machine learning) OR TI=(artificial intelligence)) | 2561 | 2024/6/11 | Web of science |
| Query: order: -announced_date_first; size: 50; date_range: from 2014-01-01 to 2024-12-31; include_cross_list: True; terms: AND abstract="Explainable AI"; OR abstract="Interpretable AI"; OR abstract="AI transparency"; OR abstract="AI explainability"; OR abstract="AI integrability"; OR abstract=interpretable AI; OR abstract="human-centered AI"; I; OR abstract="XAI" NOT abstract=“AI workflow”; AND （abstract="AI accpetance" OR "AI adoption"OR “AI trust”） | 1182 | 2024/7/10 | Arxiv |

Table 2. Search Strategy for AI Integrability from the User Perspective

| **Search Strategy** | **Number** | **Time** | **Database** |
| --- | --- | --- | --- |
| ((workflow integration[All Fields]) OR ("process integration"[All Fields]) OR ("seamless integration"[All Fields]) OR ("AI incorporation"[All Fields]) OR ("usefulness"[Title/Abstract]) OR ("ease of use"[Title/Abstract])) AND (AI[Title/Abstract]) | 477 | 2024/7/16 | pubmed |
| ("Abstract":"workflow integration"OR "process integration" OR "Abstract":"seamless integration" OR "Abstract":"incorporation" OR "Abstract":"usefulness" OR "Abstract":"ease of use" ) AND ("Abstract":"AI") | 466 | 2024/7/17 | IEEE Xplore |
| Abstract:("workflow integration" OR "process integration" OR "seamless integration" OR "incorporation" OR "usefulness" OR "ease of use") AND Abstract:("AI" OR "artificial intelligence") | 219 | 2024/7/16 | ACM Digital Library |
| ( TITLE-ABS-KEY ("workflow integration") OR TITLE-ABS-KEY ("process integration") OR TITLE-ABS-KEY("seamless integration" ) OR TITLE-ABS-KEY ("incorporation") OR TITLE-ABS-KEY ("usefulness") OR TITLE-ABS-KEY ("ease of use" ) ) AND ( TITLE-ABS-KEY ( "AI" )) AND PUBYEAR > 2013 AND PUBYEAR < 2025 | 3044 | 2024/7/17 | Scopus |
| (AB=(workflow integration) OR AB=(process integration) OR AB=(seamless integration) OR AB=(incorporation) OR AB=(usefulness) OR AB=("ease of use") ) AND (AB=(AI)) | 2385 | 2024/7/16 | Web of science |
| Query: order: -announced_date_first; size: 50; date_range: from 2014-01-01 to 2024-12-31; include_cross_list: True; terms: OR abstract="process integration"; OR abstract=seamless integration; OR abstract=workflow integration; AND title="AI" | 1017 | 2024/7/17 | Arxiv |
